# Enhancement of musculocutaneous nerve reinnervation after vascular endothelial growth factor (VEGF) gene therapy

**DOI:** 10.1186/1471-2202-13-57

**Published:** 2012-06-06

**Authors:** Pavel Haninec, Radek Kaiser, Vladimír Bobek, Petr Dubový

**Affiliations:** 1 Department of Neurosurgery, 3rd Faculty of Medicine, Charles University, Prague, Czech Republic; 2 Department of Tumor Biology, 3rd Faculty of Medicine, Charles University, Prague, Czech Republic; 3 Department of Anatomy, Division of Neuroanatomy, Faculty of Medicine, and Central European Institute of Technology (CEITEC), Masaryk University, Kamenice 3, CZ-625 00, Brno, Czech Republic

## Abstract

**Background:**

Vascular endothelial growth factor (VEGF) is not only a potent angiogenic factor but it also promotes axonal outgrowth and proliferation of Schwann cells. The aim of the present study was to quantitatively assess reinnervation of musculocutaneous nerve (MCN) stumps using motor and primary sensory neurons after plasmid phVEGF transfection and end-to-end (ETE) or end-to-side (ETS) neurorrhaphy. The distal stump of rat transected MCN, was transfected with plasmid phVEGF, plasmid alone or treated with vehiculum and reinnervated following ETE or ETS neurorrhaphy for 2 months. The number of motor and dorsal root ganglia neurons reinnervating the MCN stump was estimated following their retrograde labeling with Fluoro-Ruby and Fluoro-Emerald. Reinnervation of the MCN stumps was assessed based on density, diameter and myelin sheath thickness of regenerated axons, grooming test and the wet weight index of the biceps brachii muscles.

**Results:**

Immunohistochemical detection under the same conditions revealed increased VEGF in the Schwann cells of the MCN stumps transfected with the plasmid phVEGF, as opposed to control stumps transfected with only the plasmid or treated with vehiculum. The MCN stumps transfected with the plasmid phVEGF were reinnervated by moderately higher numbers of motor and sensory neurons after ETE neurorrhaphy compared with control stumps. However, morphometric quality of myelinated axons, grooming test and the wet weight index were significantly better in the MCN plasmid phVEGF transfected stumps. The ETS neurorrhaphy of the MCN plasmid phVEGF transfected stumps in comparison with control stumps resulted in significant elevation of motor and sensory neurons that reinnervated the MCN. Especially noteworthy was the increased numbers of neurons that sent out collateral sprouts into the MCN stumps. Similarly to ETE neurorrhaphy, phVEGF transfection resulted in significantly higher morphometric quality of myelinated axons, behavioral test and the wet weight index of the biceps brachii muscles.

**Conclusion:**

Our results showed that plasmid phVEGF transfection of MCN stumps could induce an increase in VEGF protein in Schwann cells, which resulted in higher quality axon reinnervation after both ETE and ETS neurorrhaphy. This was also associated with a better wet weight biceps brachii muscle index and functional tests than in control rats.

## Background

The microsurgical reconstruction of an interrupted nerve is based on end-to-end neurorrhaphy of the stumps without tension. To overcome more extensive defects of peripheral nerves, autologous grafts prepared from cutaneous nerves are often used [1-3]. However, it is difficult to repair a nerve if the proximal stump is not available or the autogenous nerve grafts are insufficient for reconstruction of extensive nerve damage. Especially difficult is the treatment of proximal compartments of the brachial plexus where surgical outcomes and functional restoration of the affected arm are still very limited [4]. The situations lead to a search for alternative methods that can overcome these shortcomings.

Recently, new approaches to the reinnervation of damaged nerve have been tested in animal experiments and in clinical practice based on end-to-side neurorrhaphy. In this method of neurotization, the distal nerve stump was sutured to epineurial or perineurial window of appropriate intact nerve. This type of neurorrhaphy is based on the potential formation of collateral sproutings from axons of intact peripheral nerves [5-8]. Collateral sprouts are created from Ranvier´s nodes of intact axons at the place of application of end-to-side (ETS) neurorrhaphy. The axon collaterals grow into the denervated nerve stump and take part in a functional reinnervation of peripheral structures denervated following injury to the corresponding nerve; the process is called lateral neurotization (sprouting).

It is assumed that factors released by the cells of damaged nerves stimulate intact axons to send collateral sprouts. Activated Schwann cells, which up-regulate many axon promoting factors, play an important part in the stimulation of collateral sprouting [9,10]. For example, CNTF, insulin-like growth factors I and II (IGF I-II) released by activated Schwann cells of denervated stumps have been shown to enhance collateral sprouting from donor nerves [11-14].

Vascular endothelial growth factor (VEGF) is a potent angiogenic factor that stimulates proliferation and migration of endothelial cells, formation of new blood vessels and enhances vascular permeability [15,16]. Some experiments have demonstrated that increased levels of VEGF in a damaged nerve, by direct or plasmid delivery, support and enhance the growth of regenerating nerve fibers, probably by stimulation of Schwann cells [17] or by a combination of angiogenic, neurotrophic and neuroprotective effects [18,19].

Therefore, in the present study we investigated reinnervation of MCN stumps transfected by plasmid phVEGF and reconnected by ETE neurorrhaphy with proximal stumps or by ETS neurorrhaphy with the UN. Here, we report that phVEGF transfection increases expression of VEGF in Schwann cells of the MCN stumps and promotes axon reinnervation in both ETE and ETS neurorrhaphy models.

## Results

### Immunohistochemical detection of VEGF in the intact nerve and following injection of plasmid phVEGF

**Figure 1:**
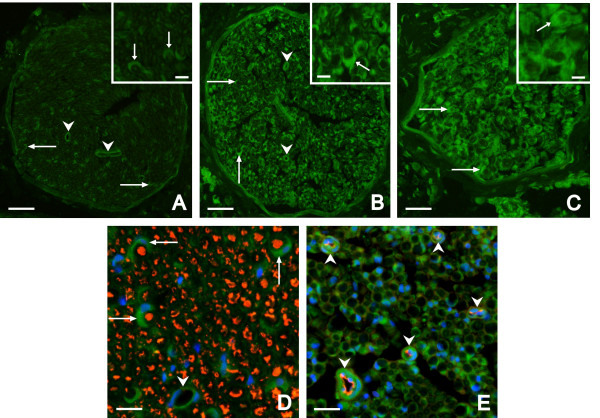
**Immunofluorescence staining for VEGF.** Immunofluorescence staining for VEGF in the cryostat sections through intact musculocutaneous nerve **(A)** and distal stump of MCN transfected with plasmid phVEGF 2 months after its end-to-end **(B)** and end-to-side **(C)** neurorrhaphy. Double immunostaining for VEGF (Alexa-488) and neurofilaments (TRITC) indicated increased VEGF staining of Schwann cells in distal stump of MCN transfected with plasmid phVEGF 2 months after its end-to-end neurorrhaphy **(D)**. Moreover, double immunostaining for VEGF (Alexa-488) and RECA (TRITC) displayed location of VEGF protein in blood vessels of transfected MCN stump 2 months after its end-to-side neurorrhaphy **(E)**. Arrows - Schwann cells; arrowheads - blood vessels. Scale bars for A-C = 90 μm, D = 20 μm, E = 30 μm, for insets = 3 μm

Transverse cryostat sections through samples of intact MCN and distal MCN stumps two months after plasmid transfection revealed VEGF immunostaining in the blood vessels and Schwann cells. Intensity of VEGF immunofluorescence was very similar in the blood vessels, but higher in Schwann cells of transfected MCN stumps compared with intact MCN (Figure 1A-C). Double immunostaining for VEGF and neurofilaments verified increased VEGF staining of Schwann cells surrounding axons in distal stump of MCN transfected with plasmid phVEGF. In addition, simultaneous immunostaining for VEGF and RECA-1 displayed VEGF level in blood vessels of transfected MCN stumps (Figure 1C, D). The results indicate that plasmid phVEGF was incorporated and increased production of VEGF protein in Schwann cells.

### Behavioral test and wet weight muscle index

The rats after ETE as well as ETS neurorrhaphy and plasmid phVEGF transfection displayed a significantly higher behavioral test score in comparison with animals treated with plasmid or vehiculum. The results of behavioral tests were significantly better in the ETE than ETS neurorrhaphy rats and the rats transfected by plasmid phVEGF (Table 1). A correlation of behavioral tests with morphometric features of regenerated axons is provided below. The biceps brachii muscle weight index reached significantly higher values in VEGF-treated animals following ETE as well as ETS neurorrhaphy compared with control animals treated with plasmid or vehiculum only. However, the wet weight muscle index was significantly higher in the rats with MCN stumps transfected with plasmid phVEGF and reconnected using ETE as opposed to ETS neurorrhaphy (Table 1).

**Table 1:** Mean scores for behavioral test and the wet weight index

	**BT ± SD**	**WW index ± SD**
**Intact MCN**	5.00 ± 0.00	1.003 ± 0.029
**Plasmid phVEGF ETE**	4.46 ± 0.56*†‡#	0.851 ± 0.078*†‡#
**Plasmid ETE**	3.42 ± 0.38*	0.774 ± 0.057*
**Vehiculum ETE**	3.42 ± 0.49*	0.760 ± 0.037*
**Plasmid phVEGF ETS**	3.83 ± 0.52*†‡	0.597 ± 0.082*†‡
**Plasmid ETS**	3.16 ± 0.26*	0.500 ± 0.069*
**Vehiculum ETS**	3.08 ± 0.21*	0.479 ± 0.079*

* indicates statistic significance (P < 0.05) when compared with intact.

† indicates statistic significance (P < 0.05) when compared with plasmid.

‡ indicates statistic significance (P < 0.05) when compared with vehiculum.

# indicates statistic significance (P < 0.05) when compared with plasmid phVEGF ETS.

Mean scores for behavioral (grooming) test (BT) and the wet weight index (WW index) of biceps brachii muscles (a ratio of ipsilateral to contralateral muscle) of intact rats and those with end-to-end (ETE) or end-to-side (ETS) neurorrhaphy after application of plasmid phVEGF, plasmid (control) and vehiculum. Six rats were used for each group.

### Quantitative analysis of retrogradely labeled dorsal root ganglia and spinal motor neurons

#### ETE neurorrhaphy

The pool of spinal motor and primary sensory neurons in the dorsal root ganglia (DRG) contributing to the innervation of the MCN from intact rats was 320 ± 34 and 1490 ± 68, respectively. Both neuronal pools are comparable with values obtained in our previous experiments [11].

The number of labeled motor and primary sensory neurons as well as their connections to the total number of labeled neurons was moderately higher in rats with MCN stumps transfected with plasmid phVEGF than in control animals with transfection of MCN stumps by plasmid or after treatment with vehiculum. However, this higher number of labeled neurons after plasmid phVEGF transfection was not statistically significant when compared with control animals (Table 2).

**Table 2:** Number of labeled motor and sensory neurons after ETE neurorrhaphy

	**Motor**	**Sensory**	**Motor/Total**	**Sensory/Total**
**Intact MCN**	308 ± 41	1549 ± 87	0.166 ± 0.010	0.864 ± 0.009
**Plasmid phVEGF ETE**	131 ± 40*	151 ± 49*	0.468 ± 0.063*	0.535 ± 0.158*
**Plasmid ETE**	97 ± 26*	121 ± 30*	0.445 ± 0.019*	0.555 ± 0.128*
**Vehiculum ETE**	84 ± 11*	105 ± 8*	0.444 ± 0.022*	0.559 ± 0.050*

* indicates statistic significance (P < 0.05) when compared with intact MCN.

Number of labeled motor and sensory neurons and their relations to the total number of labeled neurons in intact MCN and the MCN stumps after their transfection by plasmid phVEGF, plasmid alone or vehiculum treatment and ETE neurorrhaphy (n = 6 for each group). No statistically significant differences were found among experimental groups.

#### ETS neurorrhaphy

**Figure 2:**
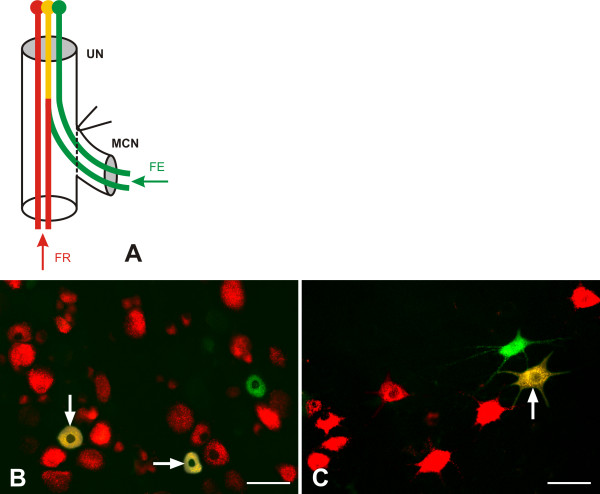
**Representative sections of C8 dorsal root ganglion and spinal cord segment.A.** A schematic drawing of double retrograde labeling of neurons following end-to-side neurorrhaphy of MCN stump with UN. FE – Fluoro-Emerald, FR – Fluoro-Ruby. **B,C.** Representative cryostat sections of C8 dorsal root ganglion **(B)** and C8 spinal cord segment **(C)** from rat 2 months after ETS neurorrhaphy of the MCN stump transfected with plasmid phVEGF illustrating retrograde labeled primary sensory and motor neurons. Red fluorescence labeled neurons with axons present only in the UN distal to ETS neurorrhaphy. Green fluorescence loaded neurons whose axons were damaged during surgical treatment and regenerated only into the MCN distal stump. Yellow fluorescence of labeled neurons (arrows) resulted from a mixture of red and green fluorescence indicating the neurons, the donor axons of which were present in the UN that had sent out collateral sprouts into the distal stump of the MCN. Scale bars = 100 μm

The axons of the UN contributed to reinnervation of MCN stumps after ETS neurorrhaphy with the UN. The UN neurons were labeled by distinct red, green and yellow (mixed) fluorescence in longitudinal sections through both the spinal cord segments (C6–Th1) and the DRG at the same levels when the FR and FE were applied into the UN and MCN stump, respectively (Figure 2). The numbers of DRG and spinal motor neurons labeled with red, green or yellow fluorescence were assessed and are summarized in Table 3.

**Table 3:** Number of labeled motor and sensory neurons after ETS neurorrhaphy

**ETS Motor**	R	G	Y	G + Y	G/Total	Y/Total	G + Y/Total
Plasmid phVEGF	128.8 ± 45.8	46.3 ± 19.0*	5.2 ± 0.8*	51.5 ± 19.3*	0.262 ± 0.088*	0.030 ± 0.007*	0.292 ± 0.089*
Plasmid	135.4 ± 34.3	9.6 ± 4.9	2.2 ± 0.8	11.8 ± 5.1*	0.071 ± 0.041	0.016 ± 0.008	0.087 ± 0.045
Vehiculum	161.3 ± 51.9	10.3 ± 2.1	1.5 ± 0.8	11.8 ± 1.6*	0.065 ± 0.025	0.009 ± 0.003	0.074 ± 0.024
**ETS Sensory**							
Plasmid phVEGF	323.2 ± 88.7	48.3 ± 18.6	28.7 ± 13.1*	77.0 ± 15.9*	0.117 ± 0.094	0.091 ± 0.070*	0.208 ± 0.080*
Plasmid	337.0 ± 57.6	33.8 ± 6.7	3.0 ± 1.7	36.8 ± 7.8	0.092 ± 0.022	0.008 ± 0.004	0.099 ± 0.024
Vehiculum	386.0 ± 34.1	35.7 ± 17.4	1.7 ± 1.2	37.3 ± 18.0	0.092 ± 0.050	0.005 ± 0.004	0.097 ± 0.033

* indicates statistically significant differences compared with the plasmid or vehiculum group (p < 0.05).

The results of number of labeled motor and sensory (DRG) neurons the axons of which are present in the UN of rats after ETS neurorrhaphy of the MCN stumps transfected by plasmid phVEGF, plasmid only or treated with vehiculum (n = 6 for each group). The motor and DRG neurons labeled with red fluorescence (R) are associated with motor and sensory axons present in the donor UN without contribution to reinnervation of the MCN stump. Green fluorescence (G) indicated neurons whose axons were damaged during surgical treatment and regenerated only into the MCN distal stump. A yellow color (Y) is the labeling of neurons that resulted from a mixture of red and green fluorescence and indicated those neurons, the donor axons of which were present in the UN and had sent out collateral sprouts into the distal stump of MCN. Therefore, G + Y labeled neurons indicated the number of neurons contributing to reinnervation of the MCN stump after ETS neurorrhaphy. The relation among G, Y and G + Y labeled neurons to total number of labeled neurons was calculated.

Red fluorescence indicated the largest number of motor and DRG neurons with axons present only in the UN distal to ETS neurorrhaphy. The numbers of red fluorescence labeled motor and sensory neurons were comparable amongst individual experimental groups without statistical significant differences.

Green fluorescence labeled UN neurons whose axons were damaged during ETS neurorrhaphy and regenerated only into the MCN distal stump. Similar numbers and proportions of green-labeled motor and DRG neurons were found in control plasmid transfected and vehiculum-treated rats while phVEGF transfection induced increased numbers and proportions of green-labeled motor and DRG neurons. However, this elevation in phVEGF transfected rats was statistically significant only in motor neurons.

Yellow fluorescence labeled neurons resulted from a mixture of red and green fluorescence indicating those neurons, the donor axons of which were present in the UN, had sent out collateral sprouts into the distal stump of the MCN. The yellow-labeled neurons were found in both the spinal ventral horn and the DRG (C6–Th1) of the operated rats. The number and proportion of yellow-labeled to total labeled motor and DRG neurons was significantly higher in phVEGF transfected compared to control rats (plasmid transfected and vehiculum-treated). The neurons with axons reinnervating into the MCN stump were labeled by green and yellow (mixed) fluorescence. The summary of green and yellow labeled DRG and motor neurons and their proportion was significantly higher in rats following phVEGF transfection of MCN stumps compared with control rats (plasmid transfection or vehiculum treatment).

### A morphometric analysis of myelinated axons regenerated into the MCN

**Figure 3:**
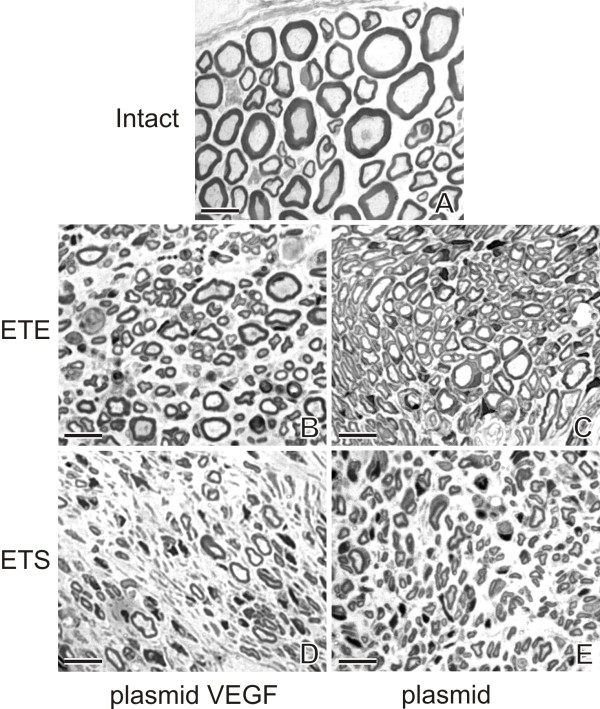
**Representative semi-thin sections through MCN.** Representative transverse semi-thin sections through the MCN of intact rat **(A)** and MCN stump transfected with plasmid phVEGF **(B, D)** or plasmid alone **(C, E)** 2 months after their end-to-end (ETE) and end-to-side (ETS) neurorrhaphy. Scale bars = 2 μm

Representative transverse sections through the intact MCN and MCN stumps 2 months after their ETE or ETS neurorrhaphy and transfected by plasmid phVEGF, plasmid and treated with vehiculum are illustrated in Figure 3.

#### ETE neurorrhaphy

Surprisingly, the number of myelinated axons per 10,000 μm² was higher in the MCN stumps of rats transfected by plasmid phVEGF or plasmid only (alone) than in the MCN removed from intact rats. However, the mean axon diameter and myelin sheath thickness were always significantly lower in all ETE treated animals compared with morphometric values of myelinated axons of the intact MCN. The MCN stumps transfected by plasmid phVEGF displayed a larger axon diameters and myelin sheath thickness than in the MCN stumps transfected by only plasmid or treated with vehiculum (Table 4).

**Table 4:** Mean density and diameter of myelinated axons and thickness of their myelin sheaths

	**Mean density of myelinated axons/10,000 μm² (n ± SD)**	**Axonal diameter (μm ± SD)**	**Myelin sheath thickness (μm ± SD)**
**Intact MCN**	138 ± 32	3.30 ± 1.23	1.20 ± 0.50
**Plasmid phVEGF ETE**	191 ± 46*†‡#	2.07 ± 1.20*†‡	0.80 ± 0.30*†‡
**Plasmid ETE**	335 ± 45*‡	1.72 ± 0.15*‡	0.80 ± 0.15*‡
**Vehiculum ETE**	110 ± 52	1.52 ± 0.71*	0.65 ± 0.25*
**Plasmid phVEGF ETS**	115 ± 40†‡	1.80 ± 0.75*†‡	0.75 ± 0.32*
**Plasmid ETS**	260 ± 25*‡	1.65 ± 0.15*‡	0.71 ± 0.15*
**Vehiculum ETS**	91 ± 40	1.45 ± 0.11*	0.70 ± 0.32*

* indicates statistic significance (P < 0.05) when compared with intact.

† indicates statistic significance (P < 0.05) when compared with plasmid.

‡ indicates statistic significance (P < 0.05) when compared with vehiculum.

# indicates statistic significance (P < 0.05) when compared with plasmid VEGF ETS.

Mean density and diameter of myelinated axons as well as thickness of the myelin sheaths in the MCN of intact rats and those with end-to-end (ETE) or end-to-side (ETS) neurorrhaphy after application of plasmid phVEGF, plasmid (control) and vehiculum. Six rats were used for each group.

#### ETS neurorrhaphy

When compared with the intact MCN, MCN stumps distal to ETS neurorrhaphy contained significantly higher density of myelinated axons per 10,000 μm² following transfection by plasmid only. The density of myelinated axons was comparable to the intact MCN when the MCN stumps were transfected with plasmid phVEGF but lower and without statistical significance after vehiculum treatment. This was because of large individual differences expressed in the SD high value.

The mean diameter of myelinated axons was significantly higher in the MCN stumps transfected with plasmid phVEGF than plasmid only or after treatment of vehiculum, but in all cases these values were significantly lower than in the intact MCN. The mean myelin sheath thickness was similar in the MCN stumps after all types of treatments and ETS neurorrhaphy, and always lower than in the intact MCN (Table 4).

#### Distribution of myelinated axon diameters

**Figure 4:**
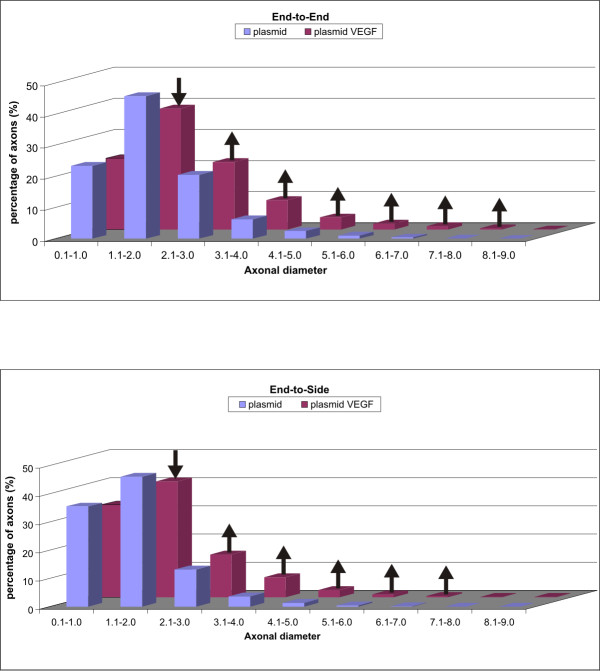
**Distribution analyses of myelinated axon diameters.** Distribution analyses of myelinated axon diameters in MCN stumps transfected with plasmid phVEGF and plasmid alone 2 months after their end-to-end and end-to-side neurorrhaphy. In comparison to transfection with plasmid alone, the phVEGF transfection induced a decreased percentage (↓) of myelinated axons with diameters from 1.1 to 2.0 μm, while there was an increased percentage (↑) in the range of 2.1 to 8.0 μm and 2.1 to 7.0 μm after end-to-end and end-to-side neurorrhaphy, respectively

Distribution analyses of myelinated axon diameters revealed that phVEGF transfection of MCN stumps induced a decreased percentage of axons in the range of 1.1 to 2.0 μm after both ETE and ETS neurorrhaphy compared with MCN stumps transfected by plasmid alone. Increased percentage of myelinated axons was found in MCN stumps transfected by phVEGF plasmid in the range of 2.1 to 8.0 μm and 2.1 to 7.0 μm after ETE and ETS neurorrhaphy, respectively (Figure 4).

## Discussion

Recent standard methods to reconnect the stumps of interrupted peripheral nerves are based on either ETE neurorrhaphy without tension or ETE with nerve graft insertion [1-3]. However, ETE neurorrhaphy is impossible if the proximal stump is not available, after avulsion of the nerve roots from the spinal cord or when graft nerve material is lacking after an extensive loss injury [20]. Therefore, clinical situations have led to a search for alternative methods including ETS neurorrhaphy based on the growth of collateral axonal sprouts. Several lines of evidence have shown that intact axon shafts send off collateral sprouts after ETS neurorrhaphy [5-7,21,22]. We have observed the formation of collateral sprouts sent off by intact sensory and motor axons of the UN into MCN stumps after ETS neurorrhaphy. In addition, retrograde labeling of the neurons using dextran conjugated with two different fluorophores (FR, FE) has also proven to be a suitable method for quantitative morphological evaluation of collateral sprouting [21,23].

It is generally accepted that creation of collateral sprouts from Ranvier’s nodes of intact axons is stimulated mainly by reactive Schwann cells of the distal stumps of severed nerve. These reactive Schwann cells produce axon promoting molecular factors [24,25], some of them, such as CNTF, basic fibroblast growth factor-2, and insulin-like growth factors I and II, are also cable of enhancing collateral sprouting [14,24,25]. Therefore, applications of axonal sprout promoting molecules together with meticulous surgical technique are believed to be key factors needed to improve results for axonal collateral sprouting after end-to-side neurorrhaphy.

It is well known that blood vessels and nerves share many similar molecular pathways during development and regeneration including creation of sprouts stimulated by VEGF [26]. It has been demonstrated in various models of nerve injury that VEGF promotes axonal outgrowth and proliferation of Schwann cells to enhance nerve reinnervation [17,19,27]. In our experimental models of ETE and ETS neurorrhaphy, we found that plasmid phVEGF transfection of the MCN stumps resulted in an elevation of VEGF protein in Schwann cells. Moreover, we demonstrated that an injection of plasmid phVEGF directly into the distal stump of the severed nerve may successfully improve its reinnervation. This easy transfection method is important issue relative to possible clinical applications.

Transfection by plasmid phVEGF increased the numbers of motor and DRG neurons, the axons of which reinnervated the distal MCN stumps after both ETE and ETS neurorrhaphy. However, an enhancement of neurons after ETE neurorrhaphy was not statistically significant compared with control animals (transfection by plasmid alone or with vehiculum treatment). This is probably because regrowth of axons after this type of reconnection is sufficiently stimulated by intrinsic cellular and molecular stimuli of the distal nerve stumps, and VEGF efficacy is not higher. In addition, no significant changes were observed in the proportion of motor and DRG neurons amongst experimental groups of animals operated on using ETE neurorrhaphy. This suggests that phVEGF transfection had no supporting effect for motor or sensory axon regrowth after this type of nerve reconnection.

In contrast to ETE neurorrhaphy, the reinnervation of the distal MCN stumps after ETS neurorrhaphy is limited to stimulation of collateral sprouting, mainly by Schwann cells [28]. Schwann cells enhanced expression of VEGF protein after plasmid phVEGF transfection and induced elevation of motor and DRG neurons that reinnervated the distal MCN stumps after ETS neurorrhaphy. It is important to note that no significant differences were obtained when we compared the proportion of motor and DRG neurons, the axons of which were present in the MCN stump (green- or yellow-labeled neurons). This result indicates that VEGF induced similar conditions for direct regrowth of motor and sensory axons and for formation of collateral sprouts from motor and sensory axons in the donor nerve without specific preference. No motor or sensory neuron preferences were found in the MCN stumps of control animals what is in agreement of our previously published results illustrating similar capacity of the motor and sensory axons to create collateral sprouts [23]. Moreover, plasmid phVEGF transfection significantly increased the number of motor and DRG neurons (green-labeled), the axons of which were regenerated directly into the MCN stumps. This suggests that VEGF may protect against the death of neurons that have lost their axon connections, as has been observed in other papers [29-31].

Although phVEGF transfection did not affect the numbers of neurons that reinnervated the MCN stumps after ETE neurorrhaphy, the increased expression of VEGF protein in Schwann cells significantly improved the morphometric quality of regenerated axons and values of behavioral test and wet weight muscle index. The axon promoting effect of VEGF transfection was also found in model of ETS neurorrhaphy, but improvement of axon diameter and myelin sheath thickness were significantly lower than in MCN stumps after ETE neurorrhaphy. However, the axon promoting effects of phVEGF transfection of MCN stumps in both neurorrhaphy models correlates with published results [17,19]. Both the increased number of neurons whose axons reinnervated, directly or by collateral sprouts, the MCN stumps after ETS neurorrhaphy and a higher quality of morphometric parameters of myelinated axons improved functional reinnervation of the biceps brachii muscles, illustrated by behavioral (grooming) test and wet weight index. Values that showed less improvement on the behavioral test and wet weight index were expected after ETS than ETE neurorrhaphy.

Surprisingly, the improvement of axon diameter and myelin sheath thickness was also found in the MCN stumps transfected only by plasmid alone when compared with vehiculum treated nerve stumps. Probably, a positive effect of plasmid transfection alone could be caused by stimulation of Schwann cell proliferation.

## Conclusions

In conclusion, our experimental results confirm that increased VEGF protein in nerve stumps, after plasmid phVEGF transfection, resulted in a higher quality of axon regeneration and functional reinnervation after both ETE and ETS neurorrhaphy.

## Methods

### Animals

Forty two adult, female, Wistar rats (Animal Breeding Facility of Masaryk University, Czech Republic) weighing approximately 250 g were used for the experiment. Experimental approaches were carried out according to the guidelines of the European Communities Council and were approved by the local ethics committee of the Faculty of Medicine, Masaryk University Brno. Six rats were used as intact controls, while the remaining thirty six animals were divided into the end-to-end (ETE, n = 18) and end-to-side (ETS, n = 18) experimental groups.

### Plasmid phVEGF preparation

Plasmid phVEGF was prepared according to published protocol [32]. Briefly, human promyelocytic leukemia HL-60 cell line was stimulated for higher production of VEGF by the tumor-promoting agent phorbol-12-myristate-13-acetate [33]. A cDNA was generated via reverse transcription using oligo(dT)23primers (Enhanced Avian RT-PCR kit; Sigma-Aldrich, St Louis, MO, USA). The specific 611–base pair fragment of the VEGF cDNA was obtained using VEGF-specific primers (5′-CCTCCGAAACCATGAACTTT-3′, 5′-GGAGGCTCCTTCCTCCTG-3′) [34,35]. The fragment was amplified using an Expand High Fidelity PCR System (Roche Diagnostics, Mannheim, Germany) and cloned via T-cloning into a pTARGET Mammalian Expression Vector (Promega, Madison, WI, USA). phVEGF165 was propagated through transformation and cultivation of Escherichia coli JM109 competent cells. The plasmid DNA was isolated from grown bacterial cultures with GenElute Endotoxin-free Plasmid Maxiprep Kit (Sigma-Aldrich) according to the directions of the manufacturer. To confirm the identity of the prepared plasmid, the VEGF-coding region from each pooled batch was re-sequenced.

### Model of end-to-end and end-to-side anastomosis and experimental treatment

All surgical treatments were performed under deep anesthesia by intraperitoneal administration of a mixture containing xylazine and ketamine. After its identification and mobilization, the MCN was cut by sharp scissors. The distal stump of the transected nerve was injected with 200 μL of phVEGF165 in PBS (DNA 10 ng/μL), vector without insert, or PBS (vehiculum) using a Hamilton micro-syringe. After 10 min, the distal MCN stump was sutured with one stitch (Ethilon, 10–0) to the proximal one (ETE neurorrhaphy) or the ulnar nerve (UN) by end-to-side anastomosis (ETS neurorrhaphy) combined with a perineurial window [11,23].

### Behavioral analysis

Behavioral analysis of active elbow flexion in the right forelimb was evaluated and scored using the grooming test [36] in the control rats and test animals two months after surgery and experimental treatment. The data were analyzed by ANOVA followed by appropriate post hoc tests (Tukey’s and Dunnett’s multiple comparisons) using Statistica 9.0 software (StatSoft, Inc.). Statistical significance was accepted at the 5% level (p < 0.05).

### Retrograde labeling of neurons

Two months after surgery, double retrograde labeling was used to identify neurons that had regenerated nerve fibers into the distal stump of the MCN after ETE or ETS neurorrhaphy and experimental treatment. The animals were reanaesthetized as described above and the MCN and/or the MCN and UN were re-exposed and transected approximately about 10 mm distal to ETE or ETS neurorrhaphy.

The UN distal stumps of ETE group of animals were inserted into the yellow pipette tips filled with 10 μl of 10% Fluoro-Ruby (FR). The nerve stumps of the UN and MCN (ETS group of animals) were inserted into the yellow pipette tips filled with 10 μl of 10% Fluoro-Ruby (FR) or Fluoro-Emerald (FE) (Molecular Probes, Inc.), respectively (Figure 2A).

Twenty minutes after fluorescent retrograde tracer application, the stumps were rinsed with phosphate buffered saline (PBS) and the wound was closed with 5/0 sutures. Six days after retrograde labeling, the rats were deeply anaesthetized by intraperitoneal injection of pentobarbital, and perfused with PBS, followed by Zamboni's fixative [37]. The C6-C8 spinal cord segments and corresponding DRG were removed and immersed in Zamboni's fixative overnight. The tissue samples were washed in 20% sucrose overnight and serial longitudinal cryostat sections (50 μm) were collected onto chrome-alum coated slides and mounted in VectaShield medium (Vector Laboratories). The sections were viewed and digitalized with a Leica DMLB fluorescence microscope equipped with a Leica DFC-480 camera (Leica Microsystems Wetzlar GmbH, Germany) using a N2.1 filter to estimate FR labeled neurons (ETE group) and a G/R filter to estimate double labeled neurons (ETS group). In addition, co-localization of both tracers retrogradely transported to the rat neurons of ETS group was also verified using individual red (filter N2.1) and green (filter I3) fluorescence profiles. Only labeled motor and DRG neurons with distinct nucleoli were counted.

The number of neurons exhibiting individual and double color of fluorescence labeling and their proportion to all labeled neurons were evaluated. The results were compared and analyzed using Mann–Whitney U-test and Statistica 9.0 software.

### Morphometric evaluation of myelinated axons in the MCN

Segments removed from MCN of intact and experimental rats before neuron retrograde labeling were fixed overnight by immersion in the fixative solution containing 4% depolymerized paraformaldehyde, 1.5% glutaraldehyde, and 10% sucrose in cacodylate buffer (0.1 M, pH 7.2). The samples were next post-fixed in 1% osmium tetroxide after washing in cacodylate buffer (0.1 M, pH 7.2) and then embedded in Durcupan (Durcupan ACM, Fluka) by the standard procedure. The transverse semi-thin sections, 80 nm thick, were stained with toluidine blue. Six randomly selected sections were digitalized under a Leica DMBL light microscope equipped with a DFC-480 digital camera at a final magnification of 6000X.

Two-dimensional disector with a circular frame was used for unbiased assessment of mean density of myelinated axons within the sampling area (10,000 μm²) and the 2D disector probes were applied for the unbiased selection of representative myelinated axons to measure their diameter as well as myelin sheath thickness [38]. A Lucia-G (Laboratory Imaging, Prague, Czech Republic) computer-assisted image analysis system was employed for measurement of pictures digitalized in the BMP format. Diameters of axons and their myelin sheath thickness were computed from cross-sectional areas, which provided the highest precision, greatest accuracy and least bias [39,40]. At least 300 myelinated axons, cut perpendicular, were measured for each MCN of the control and each experimental group with elimination of spurious and oblique profiles. The mean density of myelinated axons, axon diameters, and myelin thickness were compared among the MCN of intact and ETE and ETS experimental group rats treated with vehiculum, plasmid or phVEGF. The data were statistically evaluated using a one-way analysis of variance with post hoc comparisons of means using Statistica 9.0 software (StatSoft Inc.). Statistical significance was accepted at the 5% level (p < 0.05).

### Immunohistochemistry

Local expression of VEGF in the MCN sample removed from control and experimental rats before neuron retrograde labeling was monitored with immunohistochemistry. The nerve samples were fixed by immersion in Zamboni fixative solution overnight at 4 °C and washed in 10% phosphate-buffered sucrose for 12 h. Transverse cryostat sections (12 μm) of MCN 1–2 mm distal from ETE or ETS neurorrhaphy were washed with phosphate-buffered saline (PBS) containing 0.05% Tween 20 (PBS-TW20) and 1% bovine serum albumin (BSA) for 10 min, treated with 5% normal Donkey serum in PBS-TW20 for 30 min and incubated with 25 μl of rabbit polyclonal antibodies against VEGF (H-48, Santa Cruz, 1:200) in a humid chamber at room temperature (21–23 °C) for 12 h. The immunoreaction was visualized using Alexa-488 -conjugated and affinity purified Donkey anti-rabbit secondary antibody (Chemicon, 1:100) for 90 min at room temperature. Sections from the rat embryo were used as the positive control and sections incubated with omission of the primary antibody were used for the negative control.

A part of sections through MCN distal stumps transfected with plasmid phVEGF was simultaneously immunostained for VEGF and neurofilaments (mouse monoclonal antibody RT-97, Santa Cruz, 1:200) to prove increased VEGF staining in Schwann cells. In addition, double immunostaining for VEGF and with mouse monoclonal antibody RECA-1 (Serotec, 1:100) was used to display localization of VEGF protein in blood vessels of transfected MCN stumps. Double immunostaining was visualized using Alexa-488 (VEGF) and TRITC -conjugated (RT-97, RECA-1) and affinity purified Donkey anti-rabbit and anti-mouse secondary antibodies (Chemicon, 1:100) for 90 min at room temperature. Duijvestijn et al. [41] referred to RECA-1 as a rat pan-endothelial cell specific monoclonal antibody. Sections were mounted in a Vectashield aqueous mounting medium (Vector, CA) and analyzed using a Leica DMLB epifluorescence microscope equipped with a Leica DFC-480 camera (Leica Microsystems Wetzlar GmbH, Germany).

### Biceps brachii muscle weight analysis

The biceps brachii muscle is innervated by the MCN. When the MCN is disconnected, the biceps brachii muscle becomes atrophic, while nerve segment reinnervation results in recovery of muscle weight which is proportional to the degree of reinnervation. Wet weight analysis of the biceps brachii muscle provides an indirect evaluation of the MCN stump reinnervation.

To measure the biceps brachii muscle reinnervation, the muscles were dissected and removed from both the operated and contralateral non-operated fore limbs of rats perfused with Zamboni's fixative. The muscles were carefully wiped with filter paper and weighed immediately to compare wet weight muscles from operated and non-operated fore limb. The index of wet weight muscle was the ratio of the ipsilateral to contralateral biceps brachii muscle for each animal. Data were present as mean index ± SD.

## Competing interests

The authors declare that they have no competing interests.

## Authors´ contributions

PH conceived, designed, coordinated the study and carried out the experiments. RK participated in acquisition and analysis of the presented data. VB prepared VEGF plasmids. PD also conceived, designed, coordinated the study and wrote the manuscript. All authors gave final approval of the version to be published. All authors read and approved the final version.
